# Evaluating a digital tool for supporting people affected by breast cancer: a prospective randomized controlled trial—the ADAPT study

**DOI:** 10.1007/s00520-024-08923-3

**Published:** 2024-10-21

**Authors:** Noelle J. M. C. Vrancken Peeters, Olga Husson, Rafal Kulakowski, Emma Hainsworth, Emma Lidington, Sophie E. McGrath, Jillian Noble, Leyla Azarang, Susanne Cruickshank, Sofia Georgopoulou

**Affiliations:** 1 Netherlands Cancer Institute–Antoni van Leeuwenhoek Hospital, 1066 CX Amsterdam, The Netherlands; 2 Erasmus MC Cancer Institute, Erasmus Medical Centre, Rotterdam, The Netherlands; 3 Imperial College Health Partners, London, UK; 4 The Royal Marsden Hospital, London, UK; 5 University of Stirling, Stirling, UK

**Keywords:** Breast cancer, Patient activation, Health-related quality of life (HRQoL), Health status, Supportive care, EHealth

## Abstract

**Purpose:**

This study reports the findings from the ADAPT randomized controlled trial (RCT), concerning the impact of a digital tool for supported self-management in people affected by breast cancer on patient activation as the primary outcome, with health-related quality of life (HRQoL), and health status as secondary outcomes.

**Methods:**

Women with early-stage breast cancer were randomly assigned to standard care (control) or standard care in addition to the breast cancer digital tool (intervention). Data were collected using a demographic questionnaire, the Patient Activation Measure (PAM-13), the European Organization for Research and Treatment of Cancer Core Quality of Life Questionnaire (EORTC QLQ-C30), and the EuroQol 5-Dimension 5-Level questionnaire (EQ-5D-5L) at baseline, 6 weeks, 3 months, 6 months, and 1 year from diagnosis. Linear mixed effect model regression was used to assess the effect of the digital tool over the first year from diagnosis while correcting for intra-participant correlation.

**Results:**

A total of 166 participants were included, with 85 being randomized into the intervention. No significant differences (*p* > 0.05) in the PAM-13 scores, EORTC QLQ-C30 scales (global QoL, physical functioning, emotional functioning, pain, fatigue), and EQ-5D-5L Index between the control and intervention groups were observed. It is important to note that there was significant non-adherence within the intervention group.

**Conclusion:**

The breast cancer digital tool had no statistically significant impact on patient activation, HRQoL, and health status over time compared to standard care alone in women with early-stage breast cancer. Future research should focus on identifying and addressing barriers to digital tool engagement to improve efficacy.

Clinical trial information

The study was registered at https://clinicaltrials.gov (NCT03866655) on 7 March 2019 (https://clinicaltrials.gov/study/NCT03866655).

**Supplementary Information:**

The online version contains supplementary material available at 10.1007/s00520-024-08923-3.

## Introduction

Breast cancer is the most common cancer among women worldwide [1]. Advances in medical treatment and early detection have increased long-term survival rates, transforming breast cancer into a chronic disease [2–4]. However, despite survivors of breast cancer reporting high levels of need for support, the opportunities to receive support from health professionals have reduced over time [2]. The demand for long-term care services provision throughout the cancer continuum has been rapidly growing, but existing breast cancer interventions do not optimally satisfy the full spectrum of an individual’s care needs [5]*.* Furthermore, there is wide variability in follow-up care practices worldwide, including in the UK, where there is no standardized national approach [6]. Thus, addressing measures to support self-care among those affected by breast cancer has been receiving increased attention.

In recent years, emphasis and support for self‐management in people living with cancer have been encouraged [7–10]. Self‐management encapsulates the management of physical, social, and psychological consequences of cancer but also practical aspects as people recover from treatment. Timely recognition of the signs and symptoms of disease progression and increased understanding of ways to seek support and undertake lifestyle changes to promote health and well-being are also important components of self-management [11]. The importance of self‐management can be seen particularly in the recovery of health and well-being following cancer treatment [8].

One promising way of supporting and strengthening a person’s self-management is by using mobile health (mHealth) apps. Existing apps serve various purposes such as raising public health awareness, communicating health information, providing a platform for patient-healthcare communication, and supporting an individual’s care needs through education [12–14]. Collecting self-reported data about health needs through an app that offers a user-friendly interface can be helpful for individuals living with cancer. mHealth apps can provide health care information easily and at a minimal cost while also encouraging and motivating people to achieve treatment and health behavior goals due to immediate feedback provision [14, 15]. Previously published literature demonstrated various effects (negative and positive) of mHealth tools on the health outcomes of people affected by breast cancer [16–22]. The divergent outcomes from prior studies underscore the importance of examining the effect of mHealth tools before their broad implementation.

The primary aim of the “ADAPT study” was to evaluate the impact of a breast cancer digital tool on patient activation among early-stage breast cancer patients over time, in contrast to standard care alone. The secondary aim focused on examining the tool’s impact on health-related quality of life (HRQoL), psychological distress, health status, and National Health Service (NHS) health resource utilization over the first year from diagnosis. The breast cancer digital tool is a mHealth technology designed to support people affected by breast cancer to regain control after a diagnosis and self-manage aspects of their care. It provides needs-tailored medical information, self-monitoring of symptoms via a tracker, and functions to manage care such as an appointment calendar, modifiable question list, and a consultation recording device [23]. The breast cancer digital tool was designed collaboratively with patients and is based on the Individual and Family Self-Management Theory which postulates that individual and family health knowledge and behaviors are employed to achieve certain health outcomes through self-management. As such, individual, medical, social, and environmental factors that influence the process of self-management are central to this framework [24].

This paper presents the results related to the primary outcome of patient activation, as well as the secondary outcomes of HRQoL and health status.

## Methods

CONSORT-EHEALTH (Consolidated Standards of Reporting Trials of Electronic and Mobile HEalth Applications and onLine TeleHealth) was followed when reporting the results of this randomized controlled trial (RCT) [25]. The detailed methodology of this study is described in the study protocol, covering the breast cancer digital tool intervention, its preliminary pilot testing, participant criteria, study design, randomization, data collection methods, outcome measures, sample size estimation, and statistical analysis plan [23].

## Participants

### Eligibility criteria

Women (≥ 18 years) with primary early-stage breast cancer receiving care at one of the 4 participating sites in London (Sutton and Chelsea at the Royal Marsden Hospital, Kingston Hospital, and Croydon University Hospital) were eligible. Exclusion criteria included having already commenced anti-cancer treatment, receiving private care, inability to read or write in English, significant cognitive impairment or poor mental health (determined by the treating clinicians), lack of access to the Internet, or confirmed metastatic disease [23].

### Recruitment process

Eligible participants were continuously sampled. Research nurses reviewed clinic lists for multi-disciplinary team meetings to identify potential participants. Eligible participants were approached in person in a clinic or over the phone if there were no further clinic appointments before the start of treatment.

## Study design and methods

### Study design

The ADAPT study was a multi-center, individually randomized, parallel-controlled trial. Participants were randomly assigned (1:1) to the intervention or control arm. The control group received standard care, and the intervention group received standard care in addition to the breast cancer digital tool. Randomization was stratified by age (< 60 and ≥ 60) and center. The Institute of Cancer Research Clinical Trials and Statistics Unit (ICR-CTSU) Randomization Service generated the randomization sequence and allocated the group by phone. Due to the nature of the digital tool, it was not possible to blind participants or the healthcare team [23].

### Study groups: intervention and control

The intervention was the breast cancer digital tool, accessible online or by download on a mobile phone for free. Participants in the intervention were provided with a brochure explaining the tool at enrolment and received a unique sign-up code. A researcher followed up with participants by phone within two weeks to answer questions and troubleshoot any technical issues. Participants in the control arm received standard care including information and links to resources that people affected by breast cancer usually receive [23].

### Questionnaire packages

Participants completed the baseline questionnaire package before starting anti-cancer treatment (T0), which included participant-reported socio-demographic information along with validated questionnaires: the Patient Activation Measure (PAM-13), The European Organization for Research and Treatment of Cancer Core Quality of Life Questionnaire (EORTC QLQ-C30), and the EuroQol 5-Dimension 5-Level questionnaire (EQ-5D-5L) [26–29]. Participants completed the self-administered questionnaire package online through the Patient Reported Outcomes Following Initial treatment and Long-term Evaluation of Survivorship (PROFILES) registry system or in hard copy [30]. Data were fully anonymized, identifiable only by the study identification number and a PROFILES number automatically generated by the system. The follow-up questionnaire package was administered at 6 weeks (T1), 3 months (T2), 6 months (T3), and 1 year (T4) from diagnosis and included the same items and scales as the baseline questionnaire package with an abbreviated section of socio-demographic questions. Participants received a reminder 5 working days before the questionnaire completion deadline [23].

### MACRO database

Clinical data were extracted from the electronic patient records (EPR) of the hospital site treating the participants using an electronic case report form (CRF) and stored digitally in a central MACRO database. The CRF was completed by designated members of the clinical or research team at each hospital site as soon as possible after enrolment and at the end of follow-up. Data were fully anonymized, identifiable only by the study identification number [23].

## Primary Outcome

### Patient activation

The PAM-13 measures patient activation which refers to the knowledge, skills, and confidence a person has to manage their health and well-being [26, 27, 31]. Each item has four responses from (1) strongly disagree to (4) strongly agree, with an additional “not applicable” option. Total scores range from 0–100. The measure views activation as a developmental process of four different levels, with the lowest scores corresponding to “not believing activation is important” (≤ 47) and the highest scores corresponding to “taking action” (≥ 67.1) [23, 26, 27, 32].

## Secondary outcomes

### HRQoL

The EORTC QLQ-C30 consists of five functional scales (physical, role, cognitive, emotional, and social), a global QoL scale, three symptom scales (fatigue, nausea and vomiting, and pain), single items assessing common symptoms (dyspnea, loss of appetite, insomnia, constipation, and diarrhea), and a single item assessing perceived financial impact. After linear transformation, all scales and single-item measures range in score from 0–100. Higher scores on the functional scales and the global QoL scale indicate better functioning and HRQoL. Higher scores on the symptom scales and items as well as the financial impact item indicate higher symptom burden/financial impact [23, 28, 33, 34].

In the current study, the analysis is exclusively focused on the global QoL, physical functioning, emotional functioning, pain, and fatigue scales, as those are considered clinically the most relevant and to minimize the likelihood of type I error.

### Health status

The EQ-5D-5L consists of questions comprising the descriptive system and the EuroQol visual analog scale (EQ VAS). The descriptive system includes five dimensions: mobility, self-care, usual activities, pain/discomfort, and anxiety/depression. Each dimension has five levels of response: no problems, slight problems, moderate problems, severe problems, and extreme problems. A five-digit code generated from the responses reflects the patients’ health status. A corresponding index value is then assigned based on a UK validation study [23, 29, 35, 36].

The EQ VAS measures a patient’s self-rated health on a 0–100 vertical visual analog scale with “The best health you can imagine” at 100 and “The worst health you can imagine” at 0 [23, 29, 36].

### Contamination and non-adherence

Potential contamination and non-adherence were assessed using a single item in each of the questionnaire packages, asking whether participants had used any applications or websites (including the breast cancer digital tool) since their diagnosis to support their care. The item contained a free text box where participants could name the applications or websites they had used [23].

## Statistical analysis

Outcomes were analyzed by per-protocol analysis, due to the unavailability of MACRO data from participants who withdrew at baseline from the study (*n* = 22). Data are reported descriptively at each time point. Mean and standard deviation or median and range are reported for continuous outcomes, and frequency and percent are reported for categorical outcomes. Depending on the nature of the variable, *t*-tests or chi-square tests were used to compare baseline characteristics between the intervention and control groups [23].

### Covariates

Potential covariates considered included participant, disease, and treatment characteristics. Data collected from the EPR included tumor type, staging, date of diagnosis, date of birth, treatments received, hospitalizations, and GP registration. Covariates from the questionnaire package included single-item socio-demographic questions such as age, income, marital status, employment status, education level, baseline patient activation level, baseline QoL, baseline distress level, and comorbidities [23].

### Linear mixed effect model

To assess the effect of the breast cancer digital tool on patient activation, HRQoL, and health status over time, a multilevel approach was necessary as the longitudinal nature of the data induces correlation among the repeated measurements from the same participant over time. Therefore, linear mixed effect models were used to analyze the seven outcomes (PAM-13, EQ-5D-5L Index, and EORTC QLQ-C30 global QoL, physical functioning, emotional functioning, pain, and fatigue). In all seven models, time was specified as a categorical covariate (timepoint), its effect was considered a fixed effect, and patient ID was the grouping factor. The models incorporated the interaction between time and the study group. The baseline effect of the study groups was not included due to the randomization process. Baseline characteristics that were significantly different between the intervention and control groups were considered as fixed effects (primary tumor stage). Corresponding effect plots were created to visualize the effect of the digital tool on patient activation, HRQoL, and health status over time for a participant with the most common baseline characteristics (primary tumor stage; T1). A two-sided *p*-value of 0.05 was considered statistically significant. All statistical analyses were performed using R version 4.4.2 [37–39].

### Missing data

Missing data from multi-item scales were handled according to the guidelines of the corresponding questionnaires [26–29]. If guidelines were not available, items were mean-imputed if at least half of the items from the scale were answered. Descriptive statistics were based on a complete case analysis [23].

## Results

Of the 188 participants enrolled in the study, 22 withdrew at baseline (12%). A total of 166 participants were included in the statistical analysis; 85 were randomized into the intervention group and 81 into the control group.

### Participant characteristics

Baseline characteristics (T0) did not differ significantly between participants in the intervention and control groups, except for the primary tumor stage (*p* = 0.035, Table 1).

**Table 1:** Baseline characteristics of the study population

Variables	Control (*N* = 81)	Intervention (*N* = 85)	*p*-value
Age			
Mean (SD)	56.0 (10.4)	55.8 (12.1)	0.909
Median [min, max]	55.0 [32.0, 79.0]	56.0 [29.0, 83.0]	
Education level,* n* (%)			
Low (primary or secondary school)	14 (17.3%)	18 (21.2%)	0.508
Medium (vocational and/or college diploma)	40 (49.4%)	34 (40.0%)	
High (under- and/or post-graduate)	27 (33.3%)	32 (37.6%)	
Missing	0 (0%)	1 (1.2%)	
Partner,* n* (%)			
Yes	62 (76.5%)	64 (75.3%)	0.995
No	19 (23.5%)	21 (24.7%)	
Children,* n* (%)			
Yes	19 (23.5%)	23 (27.1%)	0.723
No	62 (76.5%)	62 (72.9%)	
Worked,* n* (%)			
Worked	47 (58.0%)	47 (55.3%)	0.765
Never worked	32 (39.5%)	37 (43.5%)	
Missing	2 (2.5%)	1 (1.2%)	
Comorbidities,* n* (%)			
None	25 (30.9%)	29 (34.1%)	0.842
1	26 (32.1%)	28 (32.9%)	
2 or more	29 (35.8%)	27 (31.8%)	
Missing	1 (1.2%)	1 (1.2%)	
Ethnicity,* n* (%)			
Non-White	21 (25.9%)	24 (28.2%)	0.945
White British	52 (64.2%)	53 (62.4%)	
White Irish and White other	8 (9.9%)	8 (9.4%)	
Breast surgery,* n* (%)			
None	1 (1.2%)	0 (0%)	0.751
Wide local excision	53 (65.4%)	51 (60.0%)	
Mastectomy	17 (21.0%)	19 (22.4%)	
Mammoplasty	8 (9.9%)	9 (10.6%)	
Missing	2 (2.5%)	6 (7.1%)	
Chemotherapy,* n* (%)			
No	0 (0%)	2 (2.4%)	0.498
Yes	81 (100%)	83 (97.6%)	
Hormonal therapy,* n* (%)			
No	3 (3.7%)	0 (0%)	0.227
Yes	78 (96.3%)	85 (100%)	
Radiotherapy,* n* (%)			
No	2 (2.5%)	1 (1.2%)	0.966
Yes	79 (97.5%)	84 (98.8%)	
Primary tumor stage, *n* (%)			
Tis	0 (0%)	7 (8.2%)	**0.035**
T1	43 (53.1%)	33 (38.8%)	
T2	29 (35.8%)	33 (38.8%)	
T3	5 (6.2%)	6 (7.1%)	
Missing	4 (4.9%)	6 (7.1%)	
Regional lymph nodes stage,* n* (%)			
N0	62 (76.5%)	58 (68.2%)	0.646
N1	12 (14.8%)	19 (22.4%)	
N2	2 (2.5%)	2 (2.4%)	
N3	1 (1.2%)	1 (1.2%)	
Missing	4 (4.9%)	5 (5.9%)	
Receptor status,* n* (%)			
HER2* positive	18 (22.2%)	20 (23.5%)	0.850
HR* positive	45 (55.6%)	48 (56.5%)	
Triple negative	12 (14.8%)	10 (11.8%)	
Missing	6 (7.4%)	7 (8.2%)	

HER2 positive; if HER2 is positive, HR positive; estrogen receptor + and/ or progesterone receptor + and HER2 − , triple negative; estrogen receptor − and progesterone receptor − and HER2 − . Bold indicates *p* < 0.05

*HER2* human epidermal growth factor receptor 2, *HR* hormone receptor

### Contamination and non-adherence

Approximately 20% of participants in the intervention group indicated they had used any applications or websites to support their care since their diagnosis (Table 2). Most of the participants in the control group stated they had never utilized any applications or websites for care support (Table 2).

**Table 2:** Usage of applications or websites

Timepoint	Control (*n* = 81)	Intervention (*n* = 85)
T0,* n* (%)		
Yes	2 (2.6)	1 (1.2)
No	75 (97.4)	80 (98.8)
Missing	4	4
T1,* n* (%)		
Yes	0 (0)	11 (21.6)
No	48 (100)	40 (78.4)
Missing	33	34
T2,* n* (%)		
Yes	1 (1.5)	13 (22.4)
No	67 (98.5)	45 (77.6)
Missing	13	27
T3, *n* (%)		
Yes	3 (5.6)	9 (17)
No	51 (94.4)	44 (83)
Missing	27	32
T4,* n* (%)		
Yes	1 (2.3)	9 (20)
No	42 (97.7)	36 (80)
Missing	38	40

*T0* baseline, *T1* 6 weeks, *T2* 3 months, *T3* 6 months, *T4* 1 year from diagnosis

### Patient activation, HRQoL, and health status over time

Participants in the intervention group tended to have higher PAM-13 scores and EQ-5D-5L Index scores, as well as higher scores on functional scales of the EORTC QLQ-C30 (global QoL, physical functioning, emotional functioning). Participants in the intervention group tended to score lower on symptom-oriented scales of the EORTC QLQ-C30 (pain, fatigue, Table 3).

**Table 3:** Patient activation, HRQoL, and health status over time

	T0		T1		T2		T3		T4	
	Control (*N* = 81)	Intervention (*N* = 85)	Control (*N* = 81)	Intervention (*N* = 85)	Control (*N* = 81)	Intervention (*N* = 85)	Control (*N* = 81)	Intervention (*N* = 85)	Control (*N* = 81)	Intervention (*N* = 85)
PAM-13 score										
Mean (SD)	58.8 (14.1)	59.8 (12.8)	58.5 (14.5)	62.2 (15.4)	60.9 (17.0)	62.5 (13.4)	63.3 (15.0)	63.2 (15.5)	58.8 (12.7)	61.5 (13.1)
Median [min, max]	53.2 [34.2, 100]	55.6 [36.8, 100]	53.2 [33.0, 100]	58.1 [40.7, 100]	55.6 [33.0, 100]	58.1 [38.1, 100]	61.9 [38.1, 100]	60.6 [34.2, 100]	56.9 [39.4, 100]	60.6 [39.4, 100]
Missing, *n* (%)	1 (1.2%)	0 (0%)	32 (39.5%)	33 (38.8%)	13 (16.0%)	27 (31.8%)	27 (33.3%)	32 (37.6%)	31 (38.3%)	34 (40.0%)
Global QoL*										
Mean (SD)	74.4 (19.2)	74.1 (19.6)	67.3 (19.3)	67.9 (19.5)	62.8 (21.5)	67.0 (18.9)	71.7 (14.1)	69.8 (19.2)	68.7 (20.2)	74.3 (15.7)
Median [min, max]	83.3 [25.0, 100]	75.0 [0, 100]	66.7 [25.0, 100]	66.7 [0, 100]	66.7 [0, 100]	66.7 [16.7, 100]	75.0 [33.3, 100]	75.0 [16.7, 100]	75.0 [0, 100]	75.0 [16.7, 100]
Missing, *n* (%)	2 (2.5%)	1 (1.2%)	32 (39.5%)	36 (42.4%)	14 (17.3%)	27 (31.8%)	30 (37.0%)	32 (37.6%)	32 (39.5%)	34 (40.0%)
Physical functioning										
Mean (SD)	90.8 (14.8)	94.0 (11.7)	82.1 (18.6)	82.7 (19.4)	82.6 (18.4)	83.3 (20.0)	83.6 (17.1)	79.6 (24.3)	81.8 (19.3)	87.7 (16.4)
Median [min, max]	100 [26.7, 100]	100 [20.0, 100]	86.7 [0, 100]	86.7 [20.0, 100]	86.7 [20.0, 100]	90.0 [26.7, 100]	86.7 [40.0, 100]	86.7 [13.3, 100]	86.7 [33.3, 100]	93.3 [20.0, 100]
Missing, *n* (%)	1 (1.2%)	0 (0%)	32 (39.5%)	34 (40.0%)	14 (17.3%)	27 (31.8%)	28 (34.6%)	32 (37.6%)	32 (39.5%)	34 (40.0%)
Emotional functioning										
Mean (SD)	66.6 (23.1)	68.7 (23.0)	68.3 (25.9)	73.6 (21.9)	68.2 (25.1)	72.8 (21.5)	75.2 (20.6)	76.1 (22.6)	69.4 (25.7)	76.6 (20.1)
Median [min, max]	66.7 [8.33, 100]	75.0 [0, 100]	75.0 [0, 100]	75.0 [16.7, 100]	66.7 [0, 100]	75.0 [16.7, 100]	75.0 [8.33, 100]	83.3 [16.7, 100]	75.0 [0, 100]	75.0 [25.0, 100]
Missing, *n* (%)	2 (2.5%)	1 (1.2%)	32 (39.5%)	36 (42.4%)	14 (17.3%)	27 (31.8%)	29 (35.8%)	32 (37.6%)	32 (39.5%)	34 (40.0%)
Pain										
Mean (SD)	14.8 (19.7)	10.8 (19.9)	31.0 (25.5)	23.5 (24.3)	27.4 (24.7)	21.6 (26.1)	26.3 (22.2)	25.5 (26.9)	29.9 (29.3)	20.9 (24.2)
Median [min, max]	0 [0, 66.7]	0 [0, 100]	33.3 [0, 100]	33.3 [0, 100]	33.3 [0, 100]	16.7 [0, 100]	16.7 [0, 100]	16.7 [0, 100]	16.7 [0, 100]	16.7 [0, 83.3]
Missing, *n* (%)	1 (1.2%)	0 (0%)	32 (39.5%)	34 (40.0%)	14 (17.3%)	27 (31.8%)	29 (35.8%)	32 (37.6%)	32 (39.5%)	34 (40.0%)
Fatigue										
Mean (SD)	21.9 (21.2)	19.3 (20.5)	38.8 (23.6)	37.0 (29.0)	45.1 (25.1)	40.4 (26.5)	37.8 (23.8)	39.2 (28.4)	35.4 (25.6)	32.9 (21.7)
Median [min, max]	11.1 [0, 66.7]	11.1 [0, 100]	33.3 [0, 100]	33.3 [0, 100]	44.4 [0, 100]	33.3 [0, 100]	33.3 [0, 100]	33.3 [0, 100]	33.3 [0, 100]	33.3 [0, 100]
Missing n (%)	1 (1.2%)	0 (0%)	32 (39.5%)	34 (40.0%)	14 (17.3%)	27 (31.8%)	29 (35.8%)	32 (37.6%)	32 (39.5%)	34 (40.0%)
EQ-5D-5L Index										
Mean (SD)	0.869 (0.110)	0.892 (0.104)	0.806 (0.150)	0.844 (0.117)	0.815 (0.139)	0.884 (0.127)	0.850 (0.111)	0.837 (0.169)	0.819 (0.157)	0.864 (0.139)
Median [min, max]	0.872 [0.496, 1]	0.922 [0.553, 1]	0.844 [0.364, 1]	0.859 [0.400, 1]	0.821 [0.280, 1]	0.909 [0.332, 1]	0.858 [0.460, 1]	0.874 [0.182, 1]	0.859 [0.300, 1]	0.887 [0.201, 1]
Missing, *n* (%)	1 (1.2%)	2 (2.4%)	33 (40.7%)	36 (42.4%)	13 (16.0%)	27 (31.8%)	28 (34.6%)	32 (37.6%)	31 (38.3%)	34 (40.0%)

For functional scales higher scores represent higher QoL; for symptom scales, higher scores indicate more severe symptoms

*QoL* quality of life, *T0* baseline, *T1* 6 weeks, *T2* 3 months, *T3* 6 months, *T4* 1 year from diagnosis

### Effect of digital tool usage on patient activation, HRQoL, and health status over time

No significant differences were observed in the PAM-13 scores at T2 between the intervention group and the control group (*p* = 0.252, Table 4).

**Table 4:** Effect of digital tool usage on patient activation, HRQoL, and health status over time

Variable	PAM-13 score		Global QoL		Physical functioning		Emotional functioning		Pain		Fatigue		EQ-5D-5L Index	
	Estimates	*p*	Estimates	*p*	Estimates	*p*	Estimates	*p*	Estimates	*p*	Estimates	*p*	Estimates	*p*
Intercept	60.52	** < 0.001**	76.61	** < 0.001**	93.76	** < 0.001**	67.17	** < 0.001**	13.17	** < 0.001**	18.86	** < 0.001**	0.88	** < 0.001**
T1	− 1.65	0.374	− 7.73	**0.007**	− 10.63	** < 0.001**	1.01	0.710	17.41	** < 0.001**	18.75	** < 0.001**	− 0.07	** < 0.001**
T2	1.32	0.420	− 11.09	** < 0.001**	− 8.86	** < 0.001**	0.29	0.904	13.07	** < 0.001**	23.22	** < 0.001**	− 0.06	** < 0.001**
T3	2.27	0.203	− 3.28	0.236	− 7.98	**0.001**	3.53	0.181	13.97	** < 0.001**	18.20	** < 0.001**	− 0.04	**0.020**
T4	− 1.13	0.536	− 6.25	**0.027**	− 8.41	** < 0.001**	3.18	0.238	15.81	** < 0.001**	12.94	** < 0.001**	− 0.06	**0.001**
T0: intervention	0.98	0.671	0.12	0.986	2.97	0.298	4.43	0.238	− 3.66	0.346	− 3.04	0.441	0.01	0.480
T1: intervention	5.36	**0.049**	− 1.96	0.604	0.92	0.785	5.41	0.212	− 7.08	0.135	1.03	0.828	0.04	0.099
T2: intervention	2.89	0.252	3.21	0.345	− 0.43	0.892	6.16	0.127	− 6.67	0.122	− 3.48	0.422	0.07	**0.002**
T3: intervention	2.29	0.389	− 1.67	0.649	− 4.37	0.187	6.13	0.148	− 3.55	0.442	0.46	0.921	0.00	0.949
T4: intervention	3.86	0.154	5.68	0.127	3.65	0.280	7.38	0.085	− 8.67	0.066	− 1.09	0.816	0.04	0.099
Observations	568		558		564		559		563		563		561	

Fixed effects are the effect of primary tumor stage, timepoint, and digital tool usage, and the grouping factor is participant ID with a random intercept. The reference category for the study group is the control group. Bold indicates *p* < 0.05

*QoL* quality of life, *T0* baseline, *T1* 6 weeks, *T2* 3 months, T3 6 months, T4 1 year from diagnosis

The PAM-13 score at T1 was significantly higher for participants in the intervention group compared to participants in the control group, as was the EQ-5D-5L Index at T2 (*p* = 0.049, *p* = 0.002, Table 4). There were no significant differences in EORTC QLQ-C30 scores (*p* > 0.05, Table 4).

When adding timepoint as a random effect, the intervention effect for the EQ-5D-5L Index at T2 remained statistically significant, but that did not hold for the PAM-13 score at T1 (Appendix Table 1).

### Effect plots

**Fig. 1:**
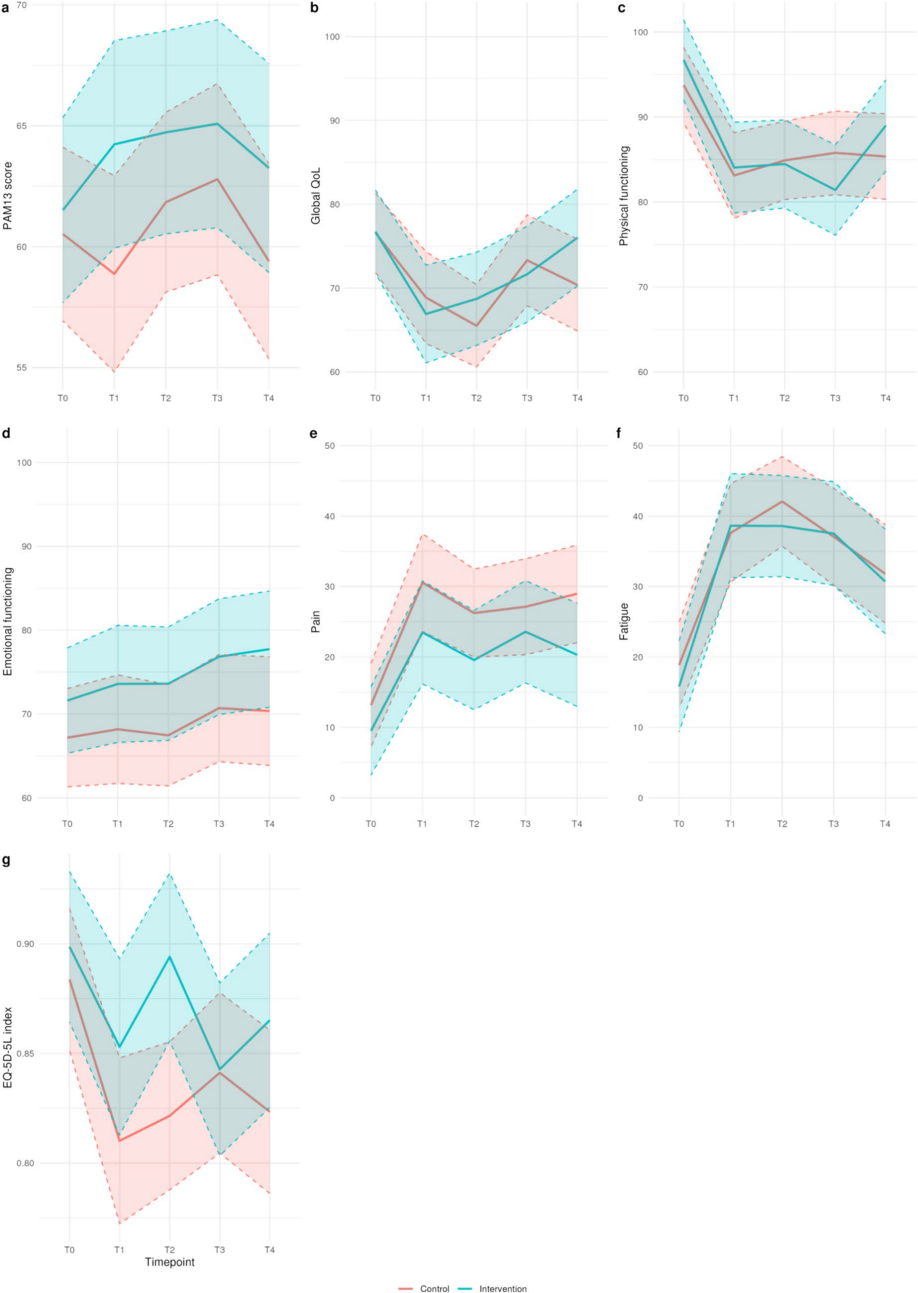
Effect plots for a participant with the most common baseline characteristics (primary tumor stage; T1). QoL, quality of life; T0, baseline; T1, 6 weeks; T2, 3 months; T3, 6 months; T4, 1 year from diagnosis. For functional scales, higher scores represent higher QoL; for symptom scales, higher scores indicate more severe symptoms

Global QoL and physical functioning tended to decrease after T0 and stabilize again after T1 (Fig. 1b, c). Symptom-orientated HRQoL scores, i.e., pain and fatigue, tended to increase after T0 and stabilize again after T1 (Fig. 1e, f). The PAM-13 score, emotional well-being, and the EQ-5D-5L Index did not show a clear trend over time (Fig. 1a, d, g).

## Discussion

This paper aimed to present the outcomes from the “ADAPT Study” concerning the impact of a breast cancer digital tool on patient activation, HRQoL, and the health status of people affected by breast cancer over time [23]. The results showed no statistically significant differences in baseline characteristics between participants in the intervention and control groups (except for the primary tumor stage), indicating effective randomization. While baseline (T0) scores for patient activation, HRQoL, and health were somewhat higher in the intervention group, these differences were not statistically significant when analyzed using linear mixed effect models. Over time, participants in the intervention group tended to score higher on patient activation, HRQoL, and health status. However, the linear mixed effect model analysis indicated that most differences did not reach statistical significance. This might be explained by the fact that only 20% of patients in the intervention group reported actively using applications or websites to support their care since their diagnosis. This suggests that a one-size-fits-all approach in mHealth apps may not enhance user engagement and, consequently, may not significantly impact patient-reported health outcomes. MHealth interventions that are more tailored to patients’ particular care needs and preferences according to their timeline, stage, and psychological status could increase user engagement and, as a result, potentially increase their effectiveness [40–42]. Currently, no clear predictors have been identified for increasing adherence to digital tools. Therefore, future research should focus on identifying demographic and cancer-related characteristics that are associated with digital tool user engagement [43].

Published literature has reported various effects of digital mHealth tools on the health outcomes of those affected by breast cancer [16–22]. In their systematic review, Jongerius et al. reported promising findings of mHealth interventions in breast cancer care management. Regarding survivorship, mHealth interventions had a positive effect through weight loss promotion, QoL improvement, and stress levels decrease. However, data on the effect of mHealth apps on psychological status were inconclusive [16]. Similarly, a recent meta-analysis assessing the effect of mHealth interventions on psychological issues experienced by women with a breast cancer diagnosis undergoing chemotherapy suggested a significant improvement in QoL but no significant changes in anxiety and depression. The authors highlighted that there was also evidence indicating reduced experience of symptoms, symptom distress, and interference and reduced supportive care needs. Additionally, a positive impact on self-efficacy, self-esteem, and emotional functioning was observed [17]. A scoping review by Cai et al. reported also promising findings about the use of mHealth apps in caring for people affected by breast cancer. Nevertheless, they conclude that there needs to be active engagement to realize their benefits, which was less than optimal in the current study [18]. Conversely, Foley et al. observed that a mHealth application providing information about breast cancer increased anxiety and depression among people affected by breast cancer undergoing surgery [21]. From the previously published literature, it may be concluded that patients’ attitudes toward mHealth tools might also be significant. While some patients may find mHealth tools helpful to their care, others may prefer not to be reminded continuously of their condition. This underscores that patient acceptance and their attitudes are of high importance in the success of mHealth tools [40].

### Strengths and limitations

An important strength of the current study is its use of longitudinal data, allowing for the examination of the effects of the digital tool on patient activation, HRQoL, and health status while correcting for time. This is important as the effect of breast cancer (diagnosis and treatment) on patient activation, HRQoL, and health status evolves over time [31, 44]. Another strength is that participants were randomly assigned to either the intervention or control group, which minimized potential bias and enhanced internal validity [45].

A limitation of the study was the high percentage of non-adherence in the intervention group. Only 20% of the patients used a digital tool since their diagnosis, potentially explaining the lack of significant differences between the two groups. Furthermore, the assessment of the intervention fidelity was limited, focusing solely on adherence, potentially leading to an oversimplified understanding of user engagement and interaction with the digital tool. To capture the complexity of digital health interactions more accurately, future research should include more robust measures of digital health use. Despite this, the sample size was adequate according to the initial sample size calculations [23]. Additionally, there was a low level of contamination in the control group, indicating that the control group was not significantly exposed to the intervention. Another notable limitation was the relatively high withdrawal rate (12%), which could potentially introduce bias. However, the absence of significant differences in baseline scores of patient activation, HRQoL, and health status suggests that the high dropout rate did not significantly impact the overall findings. Lastly, a significant limitation was the high rate of missing outcome data over time, evident even at T1, which could lead to non-response bias and underscores the need for additional research.

### Clinical implications

The findings from the current study imply that offering self-management tools directly to patients may not result in sufficient engagement to impact health outcomes significantly. To optimize user engagement with mHealth tools in the future, it may be crucial to involve healthcare professionals more actively in their integration, adapt healthcare systems to digital tools, and personalize tools to enhance efficacy [40, 42, 43, 46]. Additionally, future qualitative studies should be conducted to better understand individual attitudes, beliefs, and perceptions regarding these digital tools, aiming to improve user engagement and effectiveness. Furthermore, digital tools have the potential to transform the interaction between people with cancer and health professionals, provide support, and optimize opportunities for self-managing one’s health [22, 47, 48]. However, challenges remain. Enhancing digital and health literacy, providing education on digital tools, and addressing privacy concerns through data protection measures and open communication are essential [40]. These efforts are necessary for both people with breast cancer and the healthcare professionals providing care.

## Conclusion

Although trends toward higher health outcomes were noted, the breast cancer digital tool did not have a statistically significant impact on patient activation, HRQoL, and health status over time compared to standard care alone in women with early-stage breast cancer. However, it is important to note that there was significant non-adherence in the intervention group. To increase user engagement with digital tools in the future, subsequent research should aim to uncover and mitigate the barriers to effective digital tool engagement.

## Supplementary Information

Below is the link to the electronic supplementary material.

Supplementary file1 (DOCX 19 KB)

## Data Availability

The datasets generated during and/or analyzed during the current study are not publicly available due to privacy or ethical restrictions but are available from the corresponding author on reasonable request.
