# Supplementary material for: Evaluating a digital tool for supporting people affected by breast cancer: a prospective randomized controlled trial—the ADAPT study
Source: Support Care Cancer. 2024 Oct 21;32(11):740. doi: 10.1007/s00520-024-08923-3 (PMC11493798; doi:10.1007/s00520-024-08923-3)
Supplement: Supplementary file 1 — Supplementary file1 (DOCX 19 KB) [file 520_2024_8923_MOESM1_ESM.docx]

**APPENDIX**

Table 1. Effect of digital tool usage on patient activation, HRQoL, and health status over time

|  | **PAM-13 Score** | | **Global QoL*** | | **Physical Functioning** | | **Emotional Functioning** | | **Pain** | | **Fatigue** | | **EQ-5D-5L Index** | |
| --- | --- | --- | --- | --- | --- | --- | --- | --- | --- | --- | --- | --- | --- | --- |
| **Variable** | *Estimates* | *p* | *Estimates* | *p* | *Estimates* | *p* | *Estimates* | *p* | *Estimates* | *p* | *Estimates* | *p* | *Estimates* | *p* |
| Intercept | 60.41 | **<0.001** | 76.77 | **<0.001** | 93.22 | **<0.001** | 67.04 | **<0.001** | 12.50 | **<0.001** | 18.91 | **<0.001** | 0.88 | **<0.001** |
| T1 | -1.51 | 0.402 | -8.23 | **0.003** | -10.52 | **<0.001** | 0.98 | 0.734 | 17.49 | **<0.001** | 19.46 | **<0.001** | -0.08 | **<0.001** |
| T2 | 1.21 | 0.475 | -11.01 | **<0.001** | -8.84 | **<0.001** | 0.49 | 0.851 | 13.05 | **<0.001** | 23.48 | **<0.001** | -0.06 | **<0.001** |
| T3 | 1.64 | 0.380 | -3.11 | 0.245 | -7.70 | **0.003** | 3.63 | 0.175 | 13.89 | **<0.001** | 18.39 | **<0.001** | -0.05 | **0.009** |
| T4 | -0.94 | 0.603 | -5.59 | **0.046** | -4.44 | **<0.001** | 3.44 | 0.243 | 15.97 | **<0.001** | 12.90 | **<0.001** | -0.06 | **0.005** |
| T0: intervention | 0.90 | 0.679 | -0.03 | 0.992 | 3.43 | 0.086 | 4.38 | 0.238 | -4.18 | 0.196 | -3.24 | 0.339 | 0.02 | 0.334 |
| T1: intervention | 5.20 | 0.056 | -2.41 | 0.539 | 0.82 | 0.830 | 6.16 | 0.157 | -6.82 | 0.178 | 0.63 | 0.904 | 0.05 | 0.104 |
| T2: intervention | 2.84 | 0.300 | 2.50 | 0.471 | -1.01 | 0.772 | 5.36 | 0.196 | -6.80 | 0.129 | -2.79 | 0.551 | 0.07 | **0.002** |
| T3: intervention | 2.73 | 0.340 | -2.03 | 0.527 | -4.38 | 0.260 | 6.03 | 0.143 | -3.64 | 0.443 | -0.06 | 0.990 | 0.01 | 0.838 |
| T4: intervention | 3.65 | 0.133 | 5.72 | 0.119 | 3.58 | 0.274 | 7.05 | 0.099 | -8.93 | 0.085 | -1.55 | 0.727 | 0.04 | 0.157 |
| Observations | 568 | | 558 | | 564 | | 559 | | 563 | | 563 | | 561 | |

*QoL; quality of life. T0; baseline, T1; six weeks, T2; three months, T3; six months, T4; one year from diagnosis.

Fixed effects are the effect of primary tumor stage and digital tool usage; random effect is the effect of timepoint and grouping factor is participant ID. The reference category for the study group is the control group.

Bold indicates P < 0.05.
